# Controlling the near-field excitation of nano-antennas with phase-change materials

**DOI:** 10.3762/bjnano.4.70

**Published:** 2013-10-09

**Authors:** Tsung Sheng Kao, Yi Guo Chen, Ming Hui Hong

**Affiliations:** 1 Department of Electrical and Computer Engineering, National University of Singapore, 4 Engineering Drive 3, 117576 Singapore

**Keywords:** light localization, nano-antenna, near field, phase-change materials, plasmon coupling

## Abstract

By utilizing the strongly induced plasmon coupling between discrete nano-antennas and quantitatively controlling the crystalline proportions of an underlying Ge_2_Sb_2_Te_5_ (GST) phase-change thin layer, we show that nanoscale light localizations in the immediate proximity of plasmonic nano-antennas can be spatially positioned. Isolated energy hot-spots at a subwavelength scale can be created and adjusted across the landscape of the plasmonic system at a step resolution of λ/20. These findings introduce a new approach for nano-circuitry, bio-assay addressing and imaging applications.

## Introduction

With the rapid development of nanophotonics, increasing attention has been focused on the precise control of the highly confined optical fields on a nanoscale. To achieve the goal of controlling nanoscale light localizations, conventional focusing methods have been proved insufficient as the optical wavelength is on a much larger microscale, whereas promising approaches that use various types of nanostructures and manipulate different characteristics of an incident light beam have emerged [1–15]. For instance, a method was suggested, which is based on the use of plasmon interference where an isolated energy hot-spot can be created and positioned at an appointed location in a confined area through the active control of light illumination on nanohole arrays with optimized amplitudes and phases [1]. With other methods, it is also possible to control the near-field excitation of nano-antennas by controlling polarization singularities and subwavelength spatial phase variations at the focus of high-order beams [2]. The above approaches all provide new opportunities to control light behaviour on a nanoscale. However, they can only be performed through complex nanosystems or sophisticated manipulation of the incident light beams. Moreover, it is usually impossible to excite a single desired mode of the nanostructure and subsequently create a nanoscale energy hot-spot which may only exist in a small field of view.

**Figure 1:**
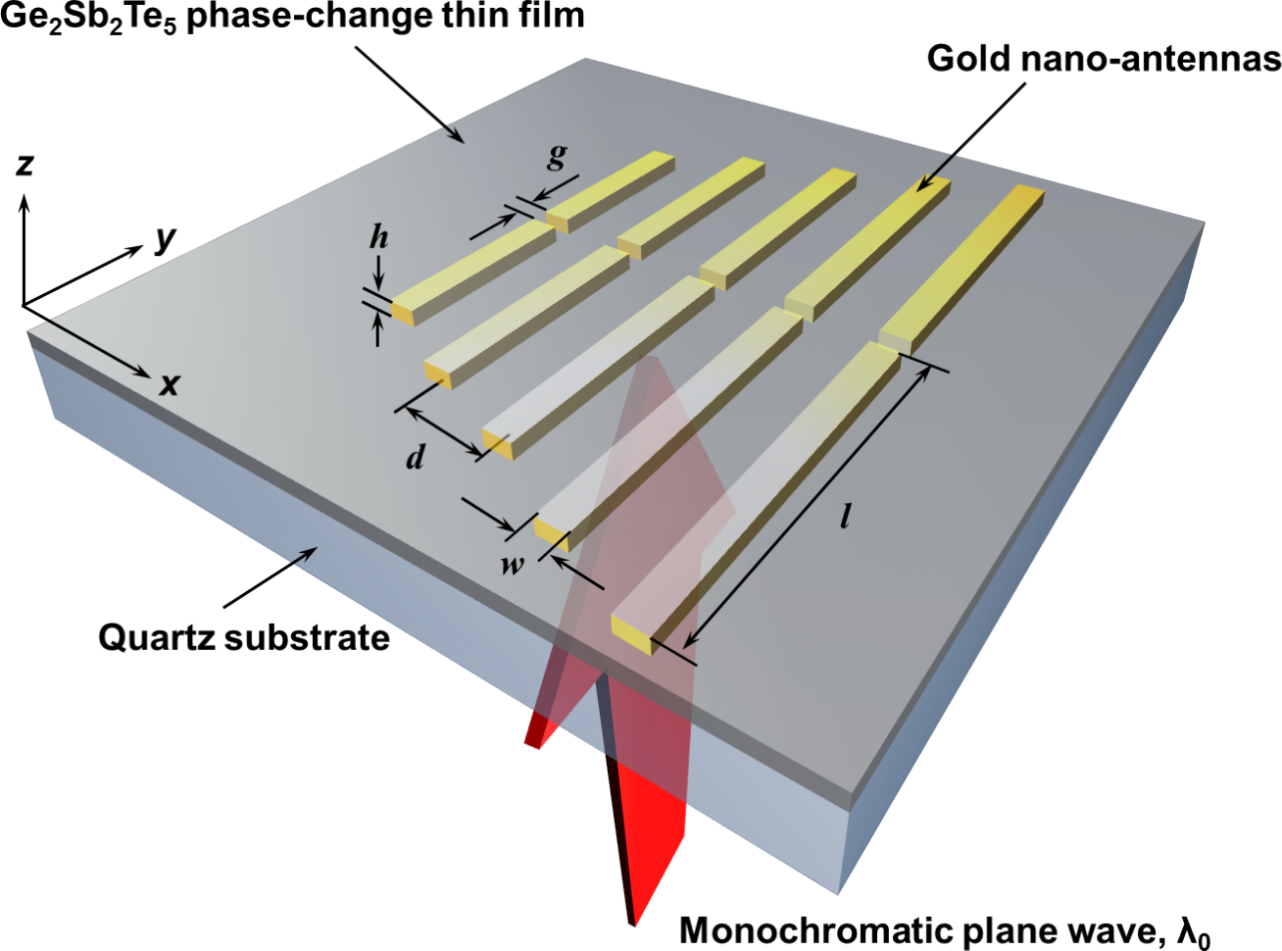
Schematic diagram of a near-field energy controllable template. With a monochromatic plane wave illumination on a nano-antenna system, the near-field intensity distributions can be selectively excited on constituent dipole nano-antennas by controlling different intermediate phases of an underneath phase-change thin film (Ge_2_Sb_2_Te_5_, GST). The hybrid plasmonic system of gold nano-antennas and phase-change materials can be fabricated on a transparent quartz substrate. The length of the antenna *l* ranges from 120 to 218 nm, while the centre-to-centre distance *d* between the nearest neighbouring antennas is 100 nm. The gap between the nano-antennas is optimized to achieve the selective excitation of the antennas and changed from 12 to 20 nm. The thickness *h* and the width *w* of antennas are both 40 nm, while the thickness of a GST thin layer is 15 nm.

Here we demonstrate a novel approach in which the constituent plasmonic resonators placed on a thin film of phase-change material can be selectively excited, generating isolated near-field energy hot-spots with selective excitation under a monochromatic plane wave illumination. Unlike other proposed techniques, our method for energy hot-spot positioning is based on a quantitative control of the crystalline proportions of the phase-change thin film rather than the complicated manipulations of an incident light beam. This makes such a near-field energy controllable template much easier to be implemented. To analyse this hybrid plasmonic system, numerical simulations were conducted by the finite-difference-time-domain (FDTD) method (FDTD Solutions 8.5, Lumerical Inc.) with realistic material parameters and Joule loss factors [16–17]. The simulation model was established and is shown in the schematic diagram Figure 1. This near-field energy controllable system consists of five gold nano-antennas with deep subwavelength spacing and an underlying thin layer of Ge_2_Sb_2_Te_5_ (GST) phase-change material supported by a transparent quartz substrate. In simulation, the gradually varying lengths of the antennas *l* are 120, 135, 150, 180, and 218 nm from the shortest to the longest one at a corresponding gap *g* of 12, 14, 14, 16, and 20 nm, respectively. These different choices in the lengths and gaps of antennas are made in order to have a comparable field enhancement on one excited nano-antenna, whereas the others remain completely unexcited. The width *w* and thickness *h* of all the antennas are 40 nm, while the centre-to-centre distance *d* between the nearest neighbouring antenna is 100 nm.

## Results and Discussion

The underlying GST thin film is a phase-change material that has been widely applied in commercial optical disks, optical switchers and phase-change memory [18–20]. GST has many attractive intrinsic properties including the large contrast of the complex refractive index between its amorphous and crystalline phases, an ultra-short tuning time (less than 30 ns), a high stability at room temperature, and a large cycle number [19,21–22]. The modulation of switching the phase states of a GST thin film can be easily accomplished by various well-developed methods, such as electric current, optical pumping and thermal stimulus [23–24]. In the near-infrared (NIR) range, a drastic change in the real part of the refractive index from the phase transition and low absorption loss allow GST to significantly alter the dielectric environment of a plasmonic system while keeping the loss manageable. Regarding the use of a GST phase-change thin film as a controllable medium, a recent research has experimentally demonstrated that the lattice resonance of a gold nanodisk array can be actively tuned in a wide range of 500 nm by controlling the GST transition phases [25].

In order to demonstrate the quantitative control of the crystalline proportions of a GST thin film, corresponding far-field spectral simulations to the experimental transmission measurements were conducted to estimate the amount of crystallized phase-change molecules. Since GST is known as a nucleation-dominated material, many small crystalline nuclei were formed first when the local temperature reaches the crystallization point of constituent phase-change molecules. Then numerous randomly-distributed small crystals joined together to form a crystalline structure [26–27]. Thus, we assume that the GST thin film at intermediate phases is composed of different proportions of amorphous and crystalline molecules, and that the refractive index of such a GST thin film can be expressed as a weighted average value of the complex indices of GST in the completely amorphous (*n*_a_(λ), *k*_a_(λ)) and crystalline (*n*_c_(λ), *k*_c_(λ)) states,

[1]
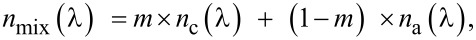


[2]
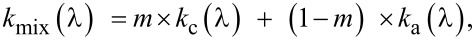


where *m* denotes the proportion of crystallized phase-change molecules ranging from 0% (amorphous) to 100% (crystalline), and the values of (*n*_a_(λ), *k*_a_(λ)) and (*n*_c_(λ), *k*_c_(λ)) are obtained from [17]. With these supposed material parameters carried out in the FDTD simulation, the corresponding refractive index values and the transmission spectra of a GST thin film at different intermediate states can be obtained by carefully selecting the proportion value *m*.

**Figure 2:**
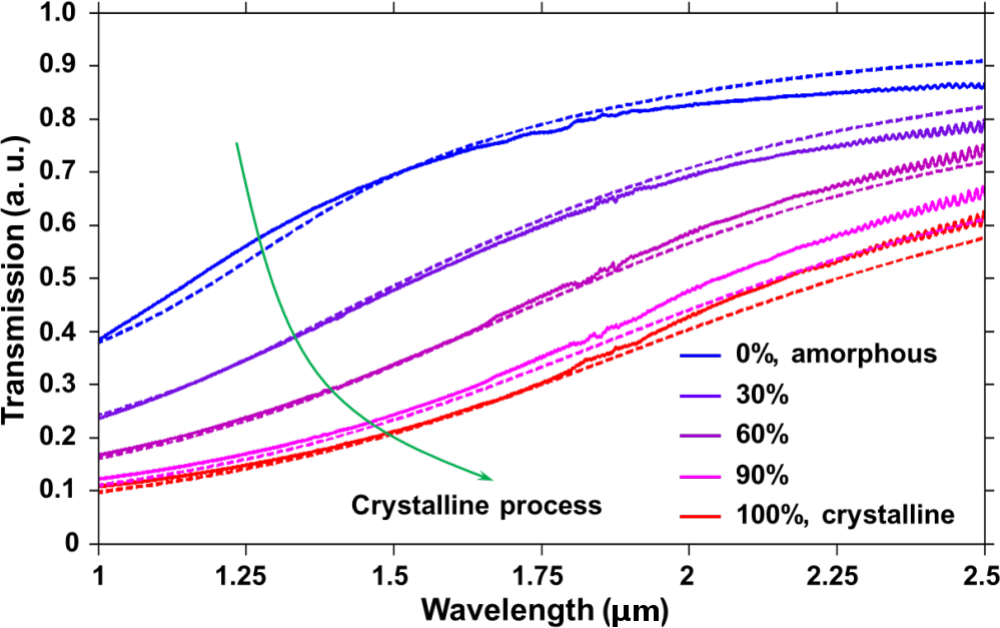
Comparison between the experimental spectra measurements (solid lines) and the simulated far-field transmission spectra (dashed lines) of a GST thin film switched at different intermediate phases. A good agreement of the spectral results shows the quantifiable phase-change modulation of a GST nano thin film at different crystalline proportions.

The phase transition of a sputtered GST phase-change thin film was performed by a homogeneous heating on a hot-plate with a crystallization temperature of 135 °C. This constant temperature was set to provide a slow phase transition rate, which facilitated a finer control of the crystalline proportions of samples. As the heating time increased, the GST thin film gradually changed from the amorphous state to the crystalline state. A UV–vis–NIR spectrophotometer (SHIMADZU, Co.) was employed to measure the transmission spectra of the GST thin film at different intermediate phases. Figure 2 shows the comparison between the experimental spectra measurements (solid lines) and the simulated far-field transmission spectra (dashed lines) of a GST thin film switched at different intermediate phases. A good agreement in both spectral results suggests that a phase-change GST thin film can be well controlled at different crystallization levels and the change of corresponding refractive index values of an underneath GST thin film may significantly alter the dielectric environment of a plasmonic system. Here a 15 nm GST thin film was used as an example.

**Figure 3:**
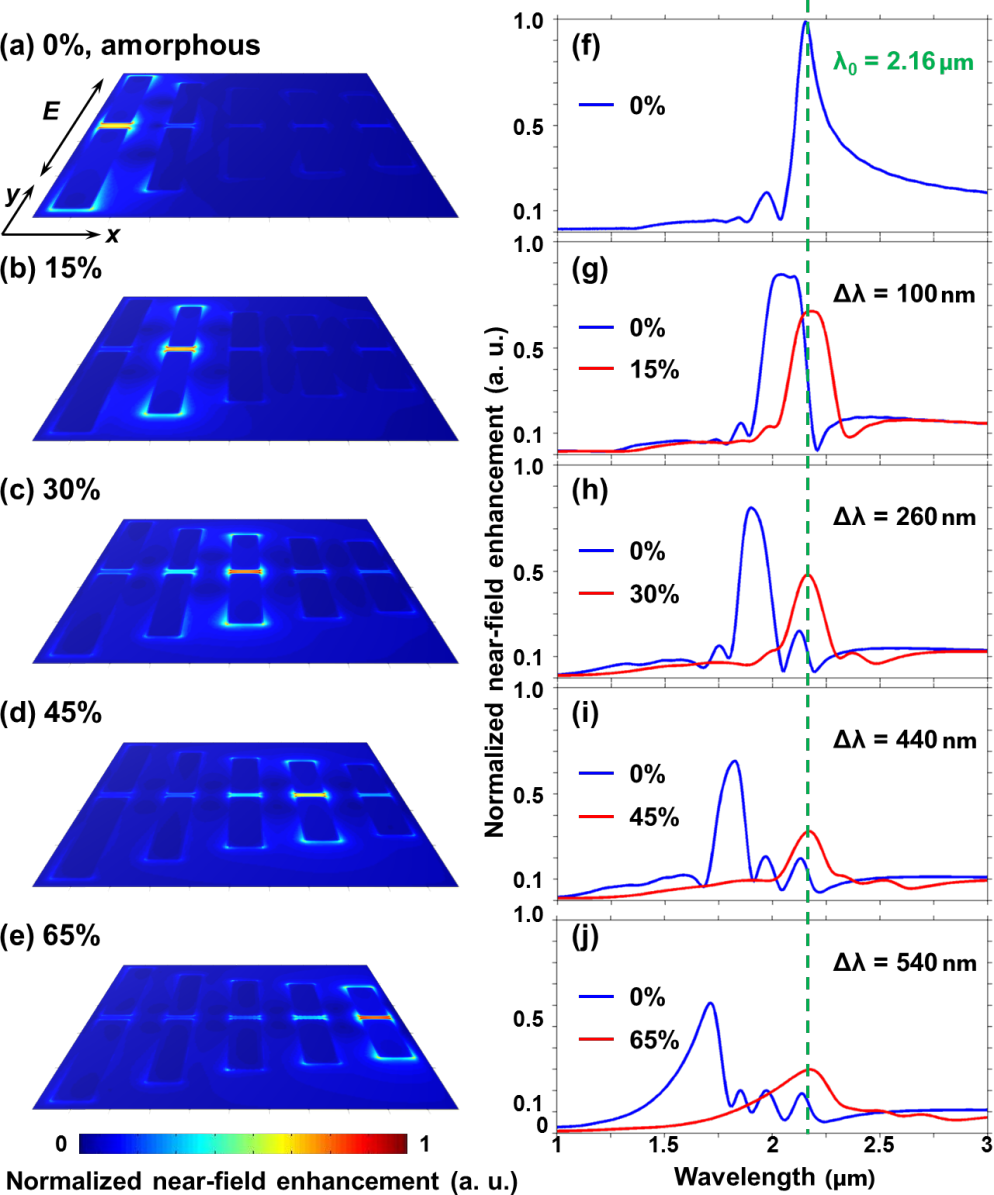
Controlling the near-field excitation of nano-antennas with a quantitative modulation on the phase transition of an underneath GST thin layer. When the crystalline proportion of the phase-change thin film is changed from the amorphous state (0%) to a portion of 65% crystallization, the nano-antennas can be selectively excited at different intermediate phases, generating nanoscale light localizations positioned on the landscape of constituent dipole nano-antennas. A plane wave is normally incident onto the array at an excitation wavelength of 2.16 µm and the polarization direction is parallel to the antennas in the *y* direction. The centre-to-centre distance between neighbouring antennas is 100 nm, giving a step resolution of λ/20 in this plasmonic nanosystem.

With the quantifiable phase-change modulation, the main characteristic features of controlling nanoscale field excitations at different transition phases are illustrated in Figure 3a–e. As shown, the nanoscale energy hot-spot is positioned on the dipole nano-antennas landscape as a function of the crystalline proportion of a GST thin film. In this plasmonic system, a plane wave is normally incident onto the array with a polarization direction parallel to the antennas in the *y* direction, and the illumination wavelength is 2.16 μm. When the underneath GST thin film is in the amorphous state, only the longest nano-antenna is excited, generating a subwavelength energy hot-spot at the gap centre of the antenna. By adjusting the proportion value *m* of crystallized phase-change molecules, the GST crystalline level with different dielectric properties is gradually changed and the excitation wavelength at the field enhancement peak of each nano-antenna can be tuned as the spectral shift shown in Figure 3g–j. Once one of the peaks corresponds to the illumination wavelength, only one corresponding nano-antenna is excited, indicating an energy hot-spot to be created at the gap centre. Through this process, the relation between the hot-spot localization and the corresponding crystalline proportion of the GST thin film can also be obtained. Thus, the nanoscale energy hot-spot can be simply repositioned from one nano-antenna to another by switching the crystalline proportion and a step resolution of λ/20 (100 nm) can be obtained much beyond the diffraction limit.

Although a clear movement and positioning of the isolated energy hot-spot can be achieved, the challenge of lower field intensity with an increase of GST crystalline proportions still needs to be addressed. These weak energy localizations may result from more metal-like portion generated when the phase-change material is changed to the crystalline state, leading to enhanced absorption and thus decreasing the transmitted light intensity. By exploiting different plasmonic resonators or changing the thickness and composites of the underlying phase-change materials, the energy loss may be reduced, increasing the feasibility to implement this near-field energy controllable template for positioning nanoscale energy hot-spots on the nanostructure landscape.

**Figure 4:**
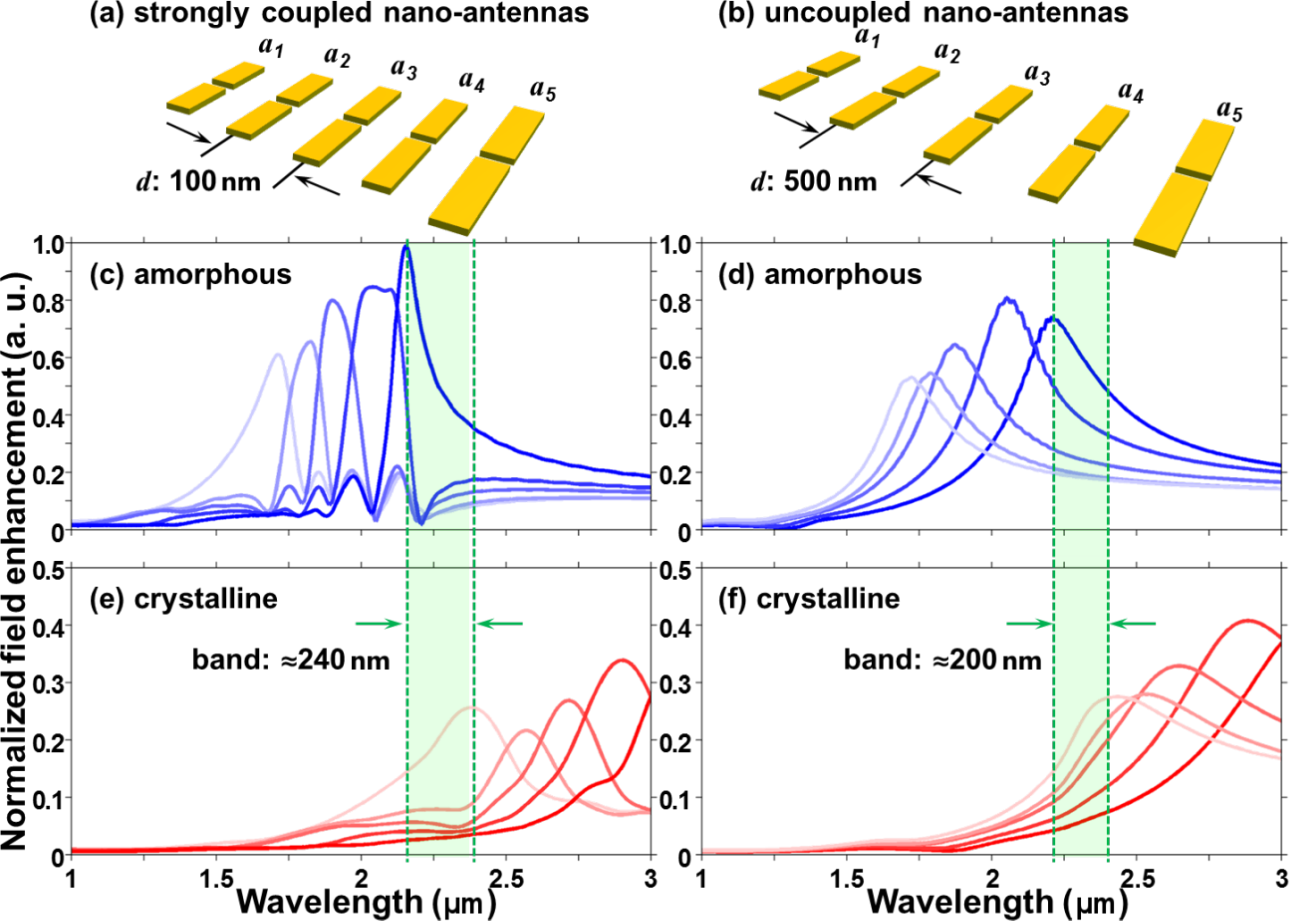
Comparisons between the strong plasmon coupling and uncoupled nano-antennas system. The antennas in the two arrays have the same geometric parameters but different centre-to-centre spacing *d*. Simulation results, (c) and (d), show a good excitation selectivity in the strong plasmon coupled nano-antenna array due to the sharp spectral features. By controlling the crystalline proportions of an underlying GST thin film, the resonance peaks of each antenna may cross a selected illumination wavelength, indicating that the antennas are individually excited. Also, a broader range of choosing feasible illumination wavelengths as the green band shows in (e) and (f) can be obtained in the strong plasmon coupling system by switching the GST completely from the amorphous state to the crystalline state.

As illustrated in [15], strong plasmon coupling between the constituent dipole antennas plays an important role in a closely packed nano-antenna array. The mutual interactions among the plasmonic resonators are hybridized and may interfere constructively at one single resonator and destructively at all the others. Thus, each antenna can be individually excited at its resonance frequency. The selective field excitation features for the coupled antenna array can also be represented in our hybridized plasmonic nanosystem. Figure 4a and b show that the arrays of nano-antennas with the same geometric parameters are placed on a GST phase-change thin film, but spatially separated with different centre-to-centre spacing *d*, while Figure 4c and d show that the calculated near-field field enhancement is modulated at the gap centres of each nano-antennas in the two arrays, respectively. In Figure 4c, at a separation distance of 100 nm, each antenna exhibits a shaper resonance peak, where all the other antennas are strongly suppressed. Compared with an uncoupled system, an array of dipole antennas with a separation distance of 500 nm shows poor excitation selectivity due to the relatively broad resonance profile as shown in Figure 4d. More importantly, in our proposed hybrid plasmonic nanosystem, a strict selection of illumination wavelengths is not necessarily required. The dipole nano-antennas can be individually excited by controlling the intermediate phases of an underneath GST thin film from the amorphous state to the crystalline state at a selected illumination wavelength. This facilitates the implementation of the new approach for us. Moreover, with a good control of the GST phase transition states, more diversities of plasmonic resonators such as nano-antennas can be included to extend the plasmonic system, allowing the nanoscale energy hot-spot to be positioned over a large area. In this hybrid plasmonic nanosystem, the strong plasmon interactions also provide a wider range of choices of feasible illumination wavelengths. The range is determined by the excitation wavelengths at the resonance peak of the shortest antenna in the crystalline state and the longest antenna in the amorphous state as the green band indicates in Figure 4e and f.

## Conclusion

In conclusion, we have demonstrated that in a hybrid plasmonic nanosystem, the constituent nano-antennas can be selectively excited with a selected illumination wavelength and near-field enhancement can be positioned at the gap centres of each antenna by controlling the intermediate phases of an underlying GST phase-change thin film. In the strong plasmon coupling nano-antenna array, a step resolution of λ/20 much beyond the diffraction limit can be obtained. Such a hybrid plasmonic system is easy to be implemented and the nanoscale energy hot-spot can be positioned in a large field of view by extending the system with different plasmon resonators, suggesting a further step toward applications such as nano-imaging, bio-assay addressing and nano-circuitry.
